# Diet in Sickness and Health

**Published:** 1896-06

**Authors:** 


DIET IN SICKNESS AND HEALTH. By MRS. ERNEST HART, formerly Student of the Faculty of Medicine of Paris, and of the London School of Medicine for Women. With an introduction by Sir Henry Thompson, F. R. C. S., M. B., London. Pp. xii.—219. Philadelphia: W. B. Saunders, 925 Walnut street. 1895.

The volume under consideration is a most useful and readable compendium of dietetics within the comprehension of the student, and especially adapted to the needs of the busy practitioner in his efforts to select and prescribe a suitable diet for the seriously afflicted entrusted to his care.

The first seven chapters, embracing forty-two pages, are devoted to a consideration of foods, their classification, properties, chemical constituents and nutrient values, and assimilation. The next thirty-four pages treat of under-feeding and over-feeding, on thinning and fattening, and dishes for the aged. Following this are three short chapters on digestion and on indigestion, in which the ascertained relation to the functions of the various organs concerned in digestion are succinctly stated. The remaining pages are given to a consideration of the diet indicated in the treatment of various diseases and diatheses. Gastric ulcer, diabetes, gout, phthisis, scurvy, typhoid fever and Bright's disease are some of the conditions for which special diets are recorded. This section of the work is peculiarly adapted to the needs of the active physician. The "bill of fare" suitable in a given disease is fully outlined and varied for each day in the week and receipts are given for the preparation of dishes mentioned.

Altogether the volume commends itself to those wanting a ready reference in selecting suitable diet where certain classes of food are aids to treatment. L. C. R.

